# Developing the Evidence Base to Inform Best Practice: A Scoping Study of Breast and Cervical Cancer Reviews in Low- and Middle-Income Countries

**DOI:** 10.1371/journal.pone.0134618

**Published:** 2015-09-01

**Authors:** Margaret M. Demment, Karen Peters, J. Andrew Dykens, Ann Dozier, Haq Nawaz, Scott McIntosh, Jennifer S. Smith, Angela Sy, Tracy Irwin, Thomas T. Fogg, Mahmooda Khaliq, Rachel Blumenfeld, Mehran Massoudi, Timothy De Ver Dye

**Affiliations:** 1 Clinical and Translational Research Institute, University of Rochester, Rochester, New York, United States of America; 2 Division of Community Health Sciences, University of Illinois at Chicago, Chicago, Illinois, United States of America; 3 Department of Family Medicine, University of Illinois at Chicago, Chicago, Illinois, United States of America; 4 Department of Public Health Sciences, University of Rochester, Rochester, New York, United States of America; 5 Department of Medicine, Griffin Hospital & Yale University-Griffin Prevention Research Center, New Haven, Connecticut, United States of America; 6 Department of Epidemiology, University of North Carolina-Chapel Hill, Chapel Hill, North Carolina, United States of America; 7 School of Nursing and Dental Hygiene, University of Hawai’i at Mānoa, Honolulu, Hawaii, United States of America; 8 Department of Obstetrics and Gynecology, University of Washington, Seattle, Washington, United States of America; 9 Department of Community and Family Health, University of South Florida, Tampa, Florida, United States of America; 10 Division of Population Health, Centers for Disease Control and Prevention, Atlanta, Georgia, United States of America; 11 Department of Obstetrics and Gynecology, University of Rochester School of Medicine and Dentistry, Rochester, New York, United States of America; State University of Maringá/Universidade Estadual de Maringá, BRAZIL

## Abstract

**Background:**

Breast and cervical cancers have emerged as major global health challenges and disproportionately lead to excess morbidity and mortality in low- and middle-income countries (LMICs) when compared to high-income countries. The objective of this paper was to highlight key findings, recommendations, and gaps in research and practice identified through a scoping study of recent reviews in breast and cervical cancer in LMICs.

**Methods:**

We conducted a scoping study based on the six-stage framework of Arskey and O’Malley. We searched PubMed, Cochrane Reviews, and CINAHL with the following inclusion criteria: 1) published between 2005-February 2015, 2) focused on breast or cervical cancer 3) focused on LMIC, 4) review article, and 5) published in English.

**Results:**

Through our systematic search, 63 out of the 94 identified cervical cancer reviews met our selection criteria and 36 of the 54 in breast cancer. Cervical cancer reviews were more likely to focus upon prevention and screening, while breast cancer reviews were more likely to focus upon treatment and survivorship. Few of the breast cancer reviews referenced research and data from LMICs themselves; cervical cancer reviews were more likely to do so. Most reviews did not include elements of the PRISMA checklist.

**Conclusion:**

Overall, a limited evidence base supports breast and cervical cancer control in LMICs. Further breast and cervical cancer prevention and control studies are necessary in LMICs.

## Introduction

As a global health priority, cancer is rapidly emerging as a visible and prevalent challenge differentially impacting low- and middle-income countries (LMICs) compared with high-income countries (HICs) [1–4]. With substantial differences between HICs and LMICs regarding health resources, environment, infrastructure, technology, and medical personnel, addressing prevention and treatment of cancer in LMIC settings may require a different evidence base [5–7]. Given the varying local resources and capacity, extrapolating from research conducted by and for HICs could lead to inappropriate conclusions and strategies [8–10]. That said, for complex non-communicable diseases like cancer, diagnosis and treatment can, similarly, be technically and medically complicated [3]. Making presumptions about the inability of LMICs to adopt best-practices identified elsewhere is equally problematic [11] since relative success in many LMICs with preventing and controlling other technically-intensive complex diseases like HIV was perhaps more successful (in some logistical aspects) than initially expected [12]. An evidence base developed within LMICs is necessary to inform optimal and effective care and successful strategies for cancer control, while avoiding erroneous assumptions and extrapolations from work done in high-income countries [13].

Though considerable regional variation exists, the cumulative probability of breast cancer for women aged 15–79 years in less developed countries in 2010 was 3.8% (95% CI: 3.4–4.1), closer to 50% higher than the rate from 1980 (2.4%; 95% CI: 2.1–2.9) [14]. The cumulative probability of cervical cancer for women aged 15–79 years in less developed countries in 2010 was 1.2% (95% CI: 0.9–1.6), slightly lower than the rate in 1980 (2.6%; 95%CI:1.7–3.3).[14] The cumulative probability of death from breast cancer for women aged 15–79 years in less developed countries in 2010 was 2.1% (95%CI: 1.7–2.3) a two-fold increase from 1980 levels (1.1%; 95% CI: 1.0–1.3) [14]. For cervical cancer the cumulative probability of death in 2010 was 0.5% (95%CI: 0.3–0.7) a third of what it was in 1980 (1.5%: 95% CI 1.0–1.9) [14]. Cancer patterns globally are anticipated to continue shifting [15], as infection-related cancers begin to decline and cancers relating to diet, lifestyle, and hormones increase, particularly in less developed countries [16].

To date, a wide range of systematic and non-systematic literature reviews have been conducted examining breast and cervical cancer control in LMICs. The purpose of this paper was to conduct a scoping study assessing the current status of published evidence for best practices across the care continuum for breast and cervical cancer in LMICs and to identify common themes and gaps that could be addressed with future research and systematic reviews. Specifically, the guiding research question was what reviews have indicated best practice for prevention, screening, diagnosis, treatment, and survivorship for breast and cervical cancer in LMICs. Researchers often undertake such “scoping studies” to examine the extent, range, and nature of research activity and identify gaps in the existing literature [17]. Given the wide range of study designs utilized in LMICs, a scoping study—essentially a “reviews of reviews”—is ideal to ascertain current evidence around breast and cervical cancer control in LMICs.

## Methods

A scoping study of breast and cervical cancer control in LMICs was conducted following the six-stage framework of Arskey and O’Malley [18] and the additional recommendations of Levac et al. [17]. This type of methodological approach attempts to systematically locate literature and classify it, but does not aim to exclude studies based on methodological quality nor to produce quantitative syntheses. Instead, scoping reviews aim to describe and summarize research findings on a specific topic and to highlight research gaps. The six stages of the framework and our specific methods are outlined below.

### 1. Research question

Following the recommendations of Levac et al. [17], we linked our research question (“*What reviews have indicated best practice for prevention*, *screening*, *diagnosis*, *treatment*, *and survivorship for breast and cervical cancer in LMICs*?*”*)with the purpose of the scoping study (“*Identify key themes*, *recommendations*, *and research/ practice gaps for breast and cervical cancer control in LMICs*”). Our primary interest was to examine the range of reviews on breast and cervical cancers in LMICs and identify priority areas for future systematic reviews and future research where the research base is lacking.

### 2. Identification of relevant studies

Searches in PubMed, Cochrane Review, and CINHAL were conducted with the following inclusion criteria: 1) published between 2005-February 2015, 2) focus on women with breast and cervical cancer, 3) focused on LMICs countries, 4) review article, and 5) published in English language. Subsequently, the search strategy for breast cancer reviews in PubMed included: ("Breast Neoplasms" (MeSH)) AND "Developing Countries" (MeSH); Filters: review; published in the last 10 years). For cervical cancer searches breast cancer was replaced by ("Uterine Cervical Neoplasms"(MeSH)). For details on MeSH subheadings please see S1 Text. In addition, “developing countries(MeSH)” was replaced by the keyword “low-income countries” and “low-income country” and unique reviews were included in the review. A few reviews [n = 3] that were not initially detected in our search because they focused on specific countries, or lacked a keyword match, but were identified by our research team as relevant to our objective were included.

### 3. Study selection

Two members of the abstraction team reviewed each manuscript for two study selection criteria: 1) a focus on cervical and breast cancer in LMICs, and 2) reflected a literature review rather than empirical research itself. A third member of the abstraction team resolved discrepancies regarding which studies to include.

### 4. Charting the data

The abstraction team consisted of eight of the authors. Two members of the abstraction team reviewed each manuscript. Items abstracted included: type of review (consensus statement—a comprehensive analysis by a panel of experts; systematic review—organized method of locating, assembling, and evaluating a body of literature; or non-systemic review—a narrative review of a body of literature but not systematically), cancer care continuum (based on the U.S. National Cancer Institutes definitions: prevention, detection, diagnosis, treatment, survivorship [19]), focus (general objective), research findings/recommendations, research/practice gaps, and limitations. A third member of the abstraction team resolved discrepancies.

### 5. Collating, summarizing, and reporting the results

We include a descriptive numerical summary of the reviews and a qualitative thematic analysis by the synthesis team to summarize findings across the cancer care continuum. The synthesis team was divided into three groups focusing on: 1) cervical cancer; 2) breast cancer; and 3) general themes. Each group reviewed the abstraction tables for themes separately and then the entire team met and discussed the themes that emerged and how best to report the results. A second round of data abstraction occurred after our synthesis meeting to capture more information about the themes identified. We assessed whether studies were based on LMICs (predominantly, partially, little, none from LMIC settings), the geographical focus of the review (global, LMIC, or a specific country), whether methods (e.g. strategy, terms, sources, inclusion/exclusion criteria) were described clearly (yes, no). Finally to summarize and collate the topic areas of each review, the abstraction team determined if a review focused on technological/behavioral interventions and implementation science themes. Reviews were categorized by topic based on if they reviewed studies regarding the theme, made recommendations based on the theme, both, or neither. Reviews were labeled behavioral or technical intervention if the authors assessed that the reviewed studies included an intervention design that focused either on changing behaviors (e.g. education) or technical (e.g. technological innovation in screening). In determining a review’s consideration of implementation science, we judged the inclusion of four major themes relying on Peters et al, 2009 [20] and 2013 [21]: governance, organizational-improvement, workforce capacity, and person- or community-centeredness (Table 1).

**Table 1:** Implementation science themes.

Implementation science theme	Key terms	Example from reviews
Governance	Policy, regulation, financing, public education, needs, constraints, barriers, and partnerships	“National policies are the platform for effective immunization programs” [71].
Organizational improvement	Implementation, quality improvement, quality assurance, performance management, guidelines, and systems strengthening.	“Where one or two dedicated staff had been designated to manage the services (coordinating facility activities, managing the screening itself, notifying women of test results, and ensuring follow-up care), services functioned much more effectively” [115].
Workforce capacity	Training, continuing education, and peer learning	“Health professional education should address surveillance for breast cancer recurrence and second primary cancers, including patient characteristics and other risk assessments” [93].
Community- or person-centeredness	Community empowerment, participation, information and education, social marketing, community-managed services, public health approaches, and community mobilization	“A key feature of a self-collected HPV testing strategy (SC-HPV) is the move of the primary screening activities from the clinic to the community” [24].

Reporting in this analysis adheres to the PRISMA guidelines for reporting results of systematic reviews (S1 Table) [22]. PRISMA stands for Preferred Reporting Items for Systematic Reviews and Meta-Analyses and is an evidence-based minimum set of items for reporting in systematic reviews and meta-analyses. The aim of the PRISMA Statement is to help authors improve the reporting of systematic reviews and meta-analyses.

### 6. Consultation

The manuscript was shared with the entire research team for feedback, insights, and editing.

## Results

**Fig 1:**
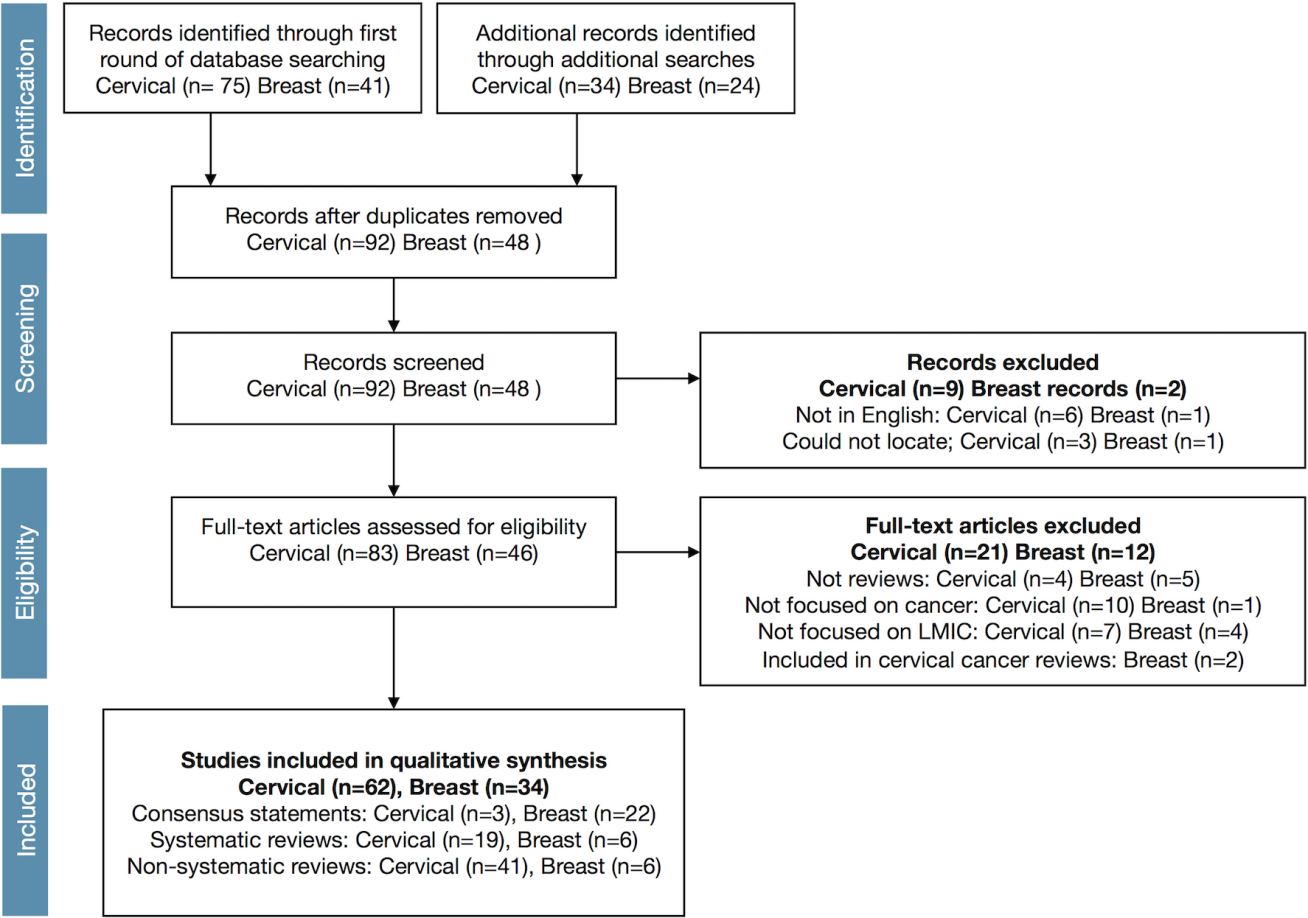
PRISMA flow diagram for review of manuscripts.

The search strategy implemented resulted in 62 reviews of cervical cancer and 34 reviews of breast cancer in LMIC settings that met our eligibility criteria (Fig 1). Cervical and breast cancer reviews differed substantially (Table 2). The cervical cancer literature reflected a substantially larger volume and distribution of both systematic [14, 23–40] and non-systematic reviews [12, 41–80], while the breast cancer literature predominantly consisted of consensus statements [81–102], with fewer systematic [103–108] or non-systematic [109–115] reviews. The cervical cancer literature also contained three consensus statements [116–118].

**Table 2:** Descriptive summary of breast and cervical cancer reviews in low- and middle-income countries (LMICs).

	Cervical cancer	Breast cancer
	n = 63	n = 36
	n (%)	n (%)
**Type of reviews**		
Consensus statements	3 (5)	23 (64)
Systematic reviews	18 (29)	6 (17)
Non-systematic reviews	43 (68)	7 (19)
**Cancer care continuum** (categories not mutually exclusive)		
Prevention	41 (65)	4 (11)
Detection	32 (51)	10 (28)
Diagnosis	15 (24)	10 (28)
Treatment	16 (25)	12 (33)
Survivorship	3 (5)	5 (14)
All	1 (2)	12 (33)
**Data from LMIC**		
Predominantly (>80%)	22 (35)	6 (17)
Partially (30–<80%)	29 (46)	6 (17)
Little (1–<30%)	12 (19)	24(67)
**Review focus on:**		
Global issues	18 (29)	1 (3)
LMIC	37 (59)	30 (83)
One region	3 (5)	2 (6)
One country	4 (6)	3 (8)
**Methods clearly described**		
Yes	14 (22)	11 (31)
No	49 (78)	25 (69)

Cervical cancer and breast cancer reviews also differed in their focus and content (Table 2). Cervical cancer reviews focused more on prevention and detection, while breast cancer reviews focused more on diagnosis, treatment, and survivorship, or more general aspects of all dimensions. Few of the breast cancer reviews referenced research and data from LMICs themselves; cervical cancer reviews were more likely to do so. Cervical cancer reviews were more globally focused, while breast cancer reviews focused more specifically on low-income regions. The wide majority of reviews in both areas (breast and cervical cancer) did not present transparent methods. Few studies reported even a subset of PRISMA elements [22].

Themes were identified if reviewed, recommended, or both, within a particular paper (Table 3). Most reviews included both (recommended and reviewed) regarding technical and behavioral interventions (Table 3). Frequently, reviews provided recommendations on a topic without first presenting an analysis of the literature on that topic. For instance, cervical cancer reviews (Table 4) tended to include recommendations around issues of governance and systems development. Similarly, both cervical and breast cancer reviews (Table 5) included recommendations around workforce capacity and person/community-centeredness. Breast cancer reviews (Table 3) were more likely to include recommendations around topics actually reviewed in all themes.

**Table 3:** Emerging thematic areas from breast and cervical cancer reviews in low- and middle-income countries (LMICs).

	Cervical cancer	Breast cancer
	n = 63	n = 36
	n (%)	n (%)
**Technical/behavioral interventions** ^**a**^		
Both reviewed and recommended	42 (67)	33 (92)
Reviewed	7 (11)	0 (0)
Recommended	11 (17)	0 (0)
Neither	3 (5)	3 (8)
**Governance** ^**b**^		
Both reviewed and recommended	19 (30)	16 (44)
Reviewed	2 (3)	0 (0)
Recommended	21 (33)	3 (8)
Neither	21 (33)	17 (47)
**Systems development** ^**c**^		
Both reviewed and recommended	15 (24)	15 (42)
Reviewed	4 (6)	0 (0)
Recommended	21 (33)	5 (14)
Neither	23 (37)	16 (44)
**Workforce capacity** ^**d**^		
Both reviewed and recommended	10 (16)	14 (39)
Reviewed	1 (2)	0 (0)
Recommended	11 (17)	6 (17)
Neither	41 (65)	16 (44)
**Person/community centeredness** ^**e**^		
Both reviewed and recommended	11 (17)	12 (33)
Reviewed	1 (2)	0 (0)
Recommended	8 (13)	3 (8)
Neither	43 (68)	21 (58)

**Note**: “Reviewed” indicates that the publication reviewed studies that pertained to this theme. “Recommended” indicates that the publication presented recommendations on the given subject.

^a^ Include reviews that assess technical or behavioral interventions

^b^ Includes reviews that discuss policy, regulation, financing, public education, needs, constraints, barriers, and partnerships.

^c^ Includes reviews that discuss considerations of organizational improvement would have commented on topics such as implementation, quality improvement, quality assurance, performance management, guidelines, and systems strengthening.

^d^ Includes reviews that discuss training, continuing education, and peer learning.

^e^ includes reviews that discuss considerations of community empowerment, participation, information and education, social marketing, community-managed services, public health approaches, and community mobilization

**Table 4:** Reviews of cervical cancer in low- and middle-income countries (LMICs).

Authors	Year	Care continuum.^a^	Focus	Data from LMICs	Trans-parent methods	Tech./ Behav. Inter-vention	Gover-nance	Health systems included	Work-force capacity	Person or comm. Centered-ness
**CONSENSUS STATEMENT**										
Bradley et al.	2005	P	LMIC	X		X	X	X	X	X
Jacob et al.	2005	T	LMIC	X		X				
Tangjitgamol et al.	2009	De, Di, T	LMIC		X	X	X	X	X	X
**SYSTEMATIC–QUALITATIVE**										
Bello et al.	2011	P	Region	X	X	X	X	X	X	X
Chamot et al.	2010	T	LMIC	X	X	X		X	X	
Cunningham et al.	2014	P	Region	X	X	X	X	X	X	X
Elit et al.	2011	De, T	LMIC	X		X	X		X	
Fesenfeld et al.	2013	P	LMIC	X	X	X	X	X		
Gravitt et al.	2011	P, De, Di	Global			X	X	X	X	X
Katz et al.	2010	P	LMIC	X		X	X	X		X
McClung & Blumenthal	2012	T	LMIC			X				
Rizvi et al.	2006	All	Global	X		X	X	X		
Sankaranarayanan et al.	2006	De	Global	X		X	X	X		
Sankaranarayanan et al.	2012	Di	Global	X		X		X		
Tsu et al.	2005	P	LMIC	X	X	X	X	X		
Williams-Brennan	2013	P	LMIC	X	X		X	X		X
**SYSTEMATIC–QUANTITATIVE**										
Arbyn et al.	2008	P	LMIC	X	X	X				
Bradford & Goodman	2013	De	LMIC	X		X				
Cuzick et al	2008	P, De, Di, T	Global	X	X	X	X	X	X	X
Datta et al.	2006	Di, T, S	Global	X		X	X	X		
Forouzanfar et al	2011	P	Global	X	X		X			
Sauvaget et al.	2011	P	LMIC	X	X	X				
**NON-SYSTEMATIC**										
Adefuye et al.	2013	P	Region	X		X				
Almonte et al.	2011	P, De	Global			X	X	X	X	X
Anorlu et al.	2007	De, Di, T	LMIC	X		X				
Batson et al.	2006	P	Global	X		X	X	X		X
Belinson et al.	2010	De	LMIC	X		X	X	X	X	X
Bharadwaj et al.	2009	P	Country	X		X	X	X	X	X
Bradford et al.	2013	P, De	LMIC	X		X	X			
Bradley et al.	2006	De, T	LMIC	X	X	X			X	X
Chirenje	2005	All	LMIC	X		X				
Cronje	2005	P, De, Di, T	Global	X		X		X	X	
Cronje	2011	De	LMIC			X		X		X
Denny	2005	P, De, Di	Global			X	X	X	X	
Denny	2012	P, De, Di, T	Global	X		X	X	X		
Denny (Best Pract Res Clin Obstet Gynaecol.)	2012	All	Global	X		X	X	X		
Denny et al.	2006	All	LMIC	X		X	X	X	X	X
Garcia-Carranca and Galvin	2007	P, De, Di	Global			X	X	X		
Hoppenot et al.	2012	P, De, Di, T	Global	X		X	X		X	
Juneja et al.	2007	De	Country	X		X		X		
Kane et al.	2012	P	LMIC	X		X				
Karimi Zarchi et al.	2009	P	LMIC	X		X				
Lowy et al.	2012	P	LMIC	X		X				
Luciani et al.	2009	P, De	Global	X		X	X	X		
Natunen et al.	2011	P	LMIC	X		X	X	X		
Parkhurst et al.	2013	P, De	LMIC	X		X	X	X		X
Patro et al.	2007	De	Country	X		X	X	X	X	
Reeler et al.	2009	P, De	LMIC	X		X	X	X		X
Safaeian et al.	2007	De	LMIC	X	X	X	X	X		
Sahasrabuddhe et al.	2011	P	LMIC	X		X	X	X		
Saleem et al.	2009	P	LMIC			X				
Sankaranarayanan et al.	2005	P, De	LMIC	X		X	X		X	
Sankaranarayanan et al.	2006	De	LMIC	X		X	X	X	X	
Sankaranarayanan et al.	2009	P	Global			X	X			
Saxena et al.	2012	De	Country	X		X	X	X		
Sherris et al.	2005	De	LMIC	X			X			X
Stanley	2006	P	LMIC			X				
Stanley	2007	P	LMIC			X			X	
Steben et al.	2012	P, De	LMIC	X		X	X	X	X	X
Tomljenovic et al.	2013	P	Global			X				
Tsu et al.	2012	P, De, Di, T	LMIC	X		X	X	X		
Woo et al.	2011	P	LMIC	X		X	X	X		X
Wright et al.	2012	P, De, Di, T	LMIC	X		X	X	X	X	

^a^ P = prevention, De = detection, Di = Diagnosis, T = Treatment, S = survivorship, All = all aspects

**Table 5:** Reviews of breast cancer in low- and middle-income countries (LMICs).

Authors	Year	Care continuum.^a^	Focus	Data from LMICs	Trans-parent methods	Tech./ Behav. Inter-vention	Gover-nance	Health systems included	Work-force capacity	Person or comm. Centered-ness
**CONSENSUS STATEMENT**										
Anderson et al.	2006	All	LMIC		X					
Anderson et al.	2015	All	LMIC			X		X	X	
Anderson et al.	2008	All	LMIC		X					
Anderson et al. (*Breast J*).	2006	All	LMIC			X	X	X	X	X
Bese et al.	2008	T	LMIC			X			X	
Cardoso et al.	2013	T, S	LMIC	X		X			X	X
Cleary et al.	2013	T	LMIC			X				X
Corbex	2012	De	LMIC			X			X	
El Saghir et al	2008	T	LMIC			X				
El Saghir et al.	2011	All	LMIC	X		X			X	X
Enui et al.	2006	T	LMIC			X	X	X		
Enui et al.	2008	T	LMIC			X				
Ganz et al.	2013	S	LMIC			X	X	X	X	X
Harford et al.	2008	All	LMIC			X	X			
Harford et al.	2011	All	LMIC	X		X	X	X	X	
Lodge & Corbex	2011	All	LMIC	X	X	X	X	X		X
Masood et al.	2008	Di	LMIC			X				
Shyyan et al.	2006	Di	LMIC		X	X	X	X	X	
Shyyan et al.l	2008	All	LMIC			X			X	
Smith et al.	2006	De	LMIC		X	X	X	X		X
Wong et al	2009	T	Region		X	X		X		
Yip et al.	2011	All	LMIC	X		X	X	X	X	
Yip et al.	2008	All	LMIC	X		X	X	X	X	
**SYSTEMATIC**										
Asadzadeh et al	2011	P, De	Country	X	X	X	X	X	X	X
Chavarri-Guerra	2012	All	Country	X	X	X	X	X	X	X
El Saghir et al	2007	Di, S	Region	X	X	X	X	X		X
Lee	2012	All	Country	X	X	X	X	X	X	
Patani et al.	2013	Di, T	Global			X		X	X	
Zelle and Baltussen	2013	De, Di, T	LMIC	X	X	X	X	X		X
**NON-SYSTEMATIC**										
Al-Foheidi et al.	2013	De	LMIC			X	X	X	X	X
Kantelhardt et al.	2008	D, T	LMIC			X	X	X		X
Keshtgar et al	2011	De, Di	LMIC			X			X	
Panieri	2012	De	LMIC			X	X			X
Romeiu	2011	P	LMIC	X						
Shetty	2011	De, Di	LMIC			X				X
Yip and Taib	2012	All	LMIC			X	X	X	X	X

^a^ P = prevention, De = detection, Di = Diagnosis, T = Treatment, S = survivorship, All = all aspects

## Discussion

Development of the evidence base for best practice around breast and cervical cancer across the cancer prevention-survivorship continuum is of primary importance to inform cancer control strategies in LMICs. In this scoping study, roughly twice as many reviews were identified relating to cervical cancer compared with breast cancer in LMICs. While cervical cancer predominates in morbidity and mortality in some areas of the world (e.g. Sub-Saharan Africa), overall breast cancer is more prevalent in LMICs than cervical cancer [14]. While *systematic* reviews of the literature from LMICs around breast and cervical cancer is lacking overall, it is particularly absent on breast cancer where only six systematic reviews were identified [16].

Evidence-based best practice arises from quality systematic reviews [118]. Production of the evidence base for both breast and cervical cancer, however, faces considerable challenges. Presently, most review papers addressing breast cancer in LMIC settings reflect consensus statements, likely the result of a lack of research in LMICs from which to form systematic (or even non-systematic) reviews. The majority of reviews around breast cancer in LMICs are not based on research generated from LMICs themselves, meaning that best practices and strategies developed in high-income regions are forming the basis, through extrapolation and perhaps erroneous assumption, for low-income regions. While most of the cervical cancer literature targeting LMICs considered for this scoping study reflected reviews that did include at least partial data generated from LMIC settings, most were not systematic reviews from which to form summaries and recommendations. The majority of reviews included in each category (breast and cervical) did not provide a transparent description of methods; hence, findings and recommendations likely could be biased, or inappropriate, based on incomplete or non-representative reviews of selected studies.

Without quality systematic reviews of breast and cervical cancer studies based on research and data from LMICs themselves or why the evidence reviewed is thought to be applicable to those countries, the evidence base will likely remain incomplete, poorly replicable, and potentially recommending inaccurate and inappropriate strategies. Additionally, reviews (especially in the cervical cancer literature) commonly over-reached the data evaluated and included recommendations on issues not reviewed elsewhere in the published paper. This over-reaching was concentrated mostly on issues of governance and systems development, essential components of cancer control but frequently not the focus of empirical research [119].

Areas of focus for the reviews included in this scoping study indeed reflect research priorities to date around breast and cervical cancer. Largely because of rapid advances in HPV testing, vaccination, and visual inspection, cervical cancer reviews are more focused on prevention and detection than other phases of the continuum; in fact, only a few cervical cancer reviews focused on survivorship. In contrast, breast cancer reviews were much less likely to focus on prevention and more likely to focus upon treatment and survivorship, topics where more global research has been completed. While the HPV vaccine has shifted the focus to prevention in cervical cancer, mammography has yet to spark this type of shift in breast cancer [120]. In terms of themes, most of the reviews for breast and cervical cancer address technical and behavioral interventions, with far less focus on implementation science such as governance, systems development, workforce capacity, and person- and community-centeredness approaches.

This scoping study has limitations. First, with many similar assessments, the search strategy could be incomplete and miss papers in other sources or that were not coded with keywords used in this search. In a few instances, reviewers identified other papers not captured by the original search strategy. Further, search terms may not have identified all reviews *relevant* to LMIC but instead only those that deal with studies in LMIC. Second, our search identified seven non-English studies that were not included in this review. Third, this study was not an exhaustive search across gray literature and all databases for review materials on breast and cervical cancer. In addition, gray literature was not the focus of this review only peer-reviewed and indexed articles as we wanted to best understand the gaps in systematic reviews on breast cancer and cervical cancer. Finally, our synthesis of the reviews was limited by the fact that most reviews were non-systematic reviews in cervical cancer and with a notable proportion of consensus statements in breast cancer without strong foundations in systematic methods. This study’s strengths, however, include a systematic methodology, a large and interdisciplinary collaborating team, and the juxtaposition of breast and cervical cancer literature together creating opportunity for comparison.

In order to provide evidence-based options in LMIC around cervical and breast cancer that are scalable, research needs to arise from LMIC settings and needs to address implementation science. Further research in low-resource settings rather than extrapolating from high-resources settings is indicated by the scoping study, particularly around breast cancer control. In addition, few of the reviews considered in this scoping study research include research that draws from implementation science or makes recommendations based on implementation science. Future research is needed across all implementation science themes, but workforce capacity and community- or person-centeredness were especially under-considered in the reviews included in this scoping study.

Demonstration is needed that shows existing therapies, diagnostic tests, and interventions developed elsewhere can be as effective and practical in LMIC areas through implementation-oriented research and assessments [121]. Complex technologies and therapies with demonstrated effectiveness elsewhere typically require different implementation strategies in LMIC regions [122–124], but can produce real population benefit [11]. Breast and cervical cancer control in LMIC regions will likely remain suboptimal with excess morbidity and mortality continually observed without LMIC-based systematic reviews of implementation strategies that can generate evidence-based recommendations. As more and more technological advances are made in both breast and cervical cancer control in LMICs, the issues around implementation science and systems development become even more critical to ensure access and appropriate resource allocation.

## Supporting Information

S1 Data Underlying data for manuscript. (XLSX)

S1 Table PRISMA checklist. (PDF)

S1 Text Keywords under MeSH headings used to identify reviews. (PDF)
