# Supplementary material for: Developing the Evidence Base to Inform Best Practice: A Scoping Study of Breast and Cervical Cancer Reviews in Low- and Middle-Income Countries
Source: PLoS One. 2015 Sep 1;10(9):e0134618. doi: 10.1371/journal.pone.0134618 (PMC4556679; doi:10.1371/journal.pone.0134618)
Supplement: S1 Text — (PDF) [file pone.0134618.s003.pdf]

Breast cancer search includes the following terms: Breast Neoplasm; Neoplasm, Breast; Neoplasms, Breast; Tumors, Breast; Breast Tumors; Breast Tumor; Tumor, Breast; Mammary Neoplasms, Human; Human Mammary Neoplasm; Human Mammary Neoplasms; Neoplasm, Human Mammary; Neoplasms, Human Mammary; Mammary Neoplasm, Human; Mammary Carcinoma, Human; Carcinoma, Human Mammary; Carcinomas, Human Mammary; Human Mammary Carcinomas; Mammary Carcinomas, Human; Human Mammary Carcinoma; Breast Cancer; Cancer, Breast; Cancer of Breast; Mammary Cancer; Malignant Neoplasm of Breast; Malignant Tumor of Breast; Breast Carcinoma; Cancer of the Breast.

Cervical cancer includes the following terms: Cervical Neoplasm, Uterine, Cervical Neoplasms, Uterine, Neoplasm, Uterine Cervical, Neoplasms, Uterine Cervical, Uterine Cervical Neoplasm, Neoplasms, Cervical, Cervical Neoplasms, Cervical Neoplasm, Neoplasm, Cervical, Neoplasms, Cervix, Cervix Neoplasms, Cervix Neoplasm, Neoplasm, Cervix, Cancer of the Uterine Cervix, Cancer of the Cervix, Cervical Cancer, Uterine Cervical Cancer, Cancer, Uterine Cervical, Cancers, Uterine Cervical, Cervical Cancer, Uterine, Cervical Cancers, Uterine, Uterine Cervical Cancers, Cancer of Cervix, Cervix Cancer, Cancer, Cervix, Cancers, Cervix.

Developing countries includes the following terms: Countries, Developing; Country, Developing; Developing Country; Least Developed Countries; Countries, Least Developed; Country, Least Developed; Developed Countries, Least; Developed Country, Least; Least Developed Country; Less-Developed Countries; Countries, Less-Developed; Country, Less-Developed; Less Developed Countries; Less-Developed Country; Under-Developed Nations; Nation, Under-Developed; Nations, Under-Developed; Under Developed Nations; Under-Developed Nation; Third-World Countries; Countries, Third-World; Country, Third-World; Third World Countries; Third-World Country; Third-World Nations; Nation, Third-World; Nations, Third-World; Third World Nations; Third-World Nation; Under-Developed Countries; Countries, Under-Developed; Country, Under-Developed; Under Developed Countries; Under-Developed Country; Developing Nations; Developing Nation; Nations, Developing; Less-Developed Nations; Less Developed Nations; Less-Developed Nation; Nation, Less-Developed; Nations, Less-Developed
